# Supplementary material for: A live online exercise program for older adults improves depression and life-space mobility: A mixed-methods pilot randomized controlled trial
Source: PLoS One. 2024 Nov 11;19(11):e0312992. doi: 10.1371/journal.pone.0312992 (PMC11554215; doi:10.1371/journal.pone.0312992)
Supplement: S3 File — (PDF) [file pone.0312992.s004.pdf]

1.0 Background and rationale

The outbreak of COVID-19, due the novel coronavirus (SARS-CoV-2), has led to a global pandemic that has drastically changed the lives of Canadians. Older adults and those with comorbidities are at higher risk of mortality due to COVID-19 (1). The high human-to-human transmission potential has resulted in public health-mandated physical distancing measures to reduce the risk of infection. Unfortunately, with this stay-at-home order, many older adults have now lost ways to connect with support networks and health and social service providers (2). Additionally, older adults are spending increased time at home and alone (2). These measures increase the risk for secondary conditions including declines in mental health (3, 4), physical health and mobility (5-7), and increased risk of social isolation (2, 3, 5).

The indirect consequences of the pandemic, such as sedentary lifestyles, may result in long-term negative health outcomes. Physical inactivity is a significant public health concern as it contributes to an increased risk of morbidities, particularly for older adults (8). There is strong evidence for the beneficial role of physical activity in offsetting or delaying age-related declines in functional capacity, quality of life, morbidity, disability, and mortality (8-11). Exercise and physical activity have been found to be effective in the alleviation of strength and mobility declines (9, 12, 13), reducing anxiety (11), reducing depression (14), and alleviating cognitive declines (8, 9). There is also a strong association between lower levels of exercise and physical activity and the incidence of social isolation and feelings of loneliness (15). Critically, exercise-based intervention trials have been shown to reduce social isolation and loneliness (16, 17). However, engagement in physical activity decreases as individuals age and this is likely being exacerbated by the COVID-19 pandemic (18). Implementing new technologies utilizing online resources may help to mitigate declines in older adults' health during and post-pandemic.

With the current mandates in place, it is important to determine a way in which we can engage older adults in physical activity while maintaining safe physical distancing. Individuals are embracing the use of technology as a viable service-delivery option for traditional interventions that are typically offered in-person (e.g., exercise, dementia care, and caregiver support) (2). We propose that there is a need for home-based workouts targeted for older persons; however, older adults do not want to engage in physical activity using online tools, such as pre-recorded videos (5). These online tools lack a sense of social connectivity, which older adults prefer (5). Therefore, we hypothesize that those older adults engaging in age-appropriate and ability-modified low-risk exercise and activity programming via live video streams from qualified instructors will participate in more physical activity.

2.0 Research questions, hypotheses, and aims

2.1 Primary question and hypothesis

Do older adults engaging in age-appropriate and ability-modified low-risk exercise and activity programming via live video streams from qualified instructors have improved levels of physical activity?

We hypothesize that older adults engaging in age-appropriate and ability-modified low-risk exercise and activity programming via live video streams from qualified instructors will have improved levels of physical activity compared to a waitlist control group.

2.2 Secondary question and hypothesis

Do older adults engaging in age-appropriate and ability-modified low-risk exercise and activity programming via live video streams from qualified instructors have improved nutritional assessment scores, life-space mobility scores and reduced anxiety, depression, and feelings of loneliness? And is an age-appropriate and ability-modified low-risk exercise and activity program via live video streams feasible for older adults?

We hypothesize that older adults engaging in age-appropriate and ability-modified low-risk exercise and activity programming via live video streams from qualified instructors will have improved nutritional assessment scores, life-space mobility scores and reduced anxiety, depression, and feelings of loneliness compared to a waitlist control group. We also hypothesize that an age-appropriate and ability-modified low-risk exercise and activity program via live video streams from qualified instructors is feasible for older adults.

3.0 Methodology

3.1 Study design

A mixed-methods design will assist us in evaluating the preliminary efficacy of the intervention in this pilot study. The mixed methods design will consist of:

1) a quantitative, two-arm randomized controlled trial (RCT) using accelerometers and questionnaires completed by study participants at baseline, following the 8-week exercise program and 8-weeks after the program to measure physical activity, nutritional status, life space mobility, loneliness, anxiety, and depression;
2) a qualitative descriptive study in which we will conduct, record, transcribe and analyze via video (Zoom), in-person narrative interviews with a purposeful sample of participants to more thoroughly understand participants' motivation, attitudes, and beliefs regarding exercise classes and their satisfaction with the program.

3.2 Study population

3.2.1 Inclusion criteria

This study will include English-speaking community-dwelling adults living in Hamilton and the surrounding areas, who are between the ages of 65 – 80 years.

3.2.2 Exclusion criteria

Potential participants will be excluded if they satisfy any of the following criteria:

1) experience any physical conditions which may prevent them from engaging in moderate-intensity physical activity including using assistive walking devices (e.g. cane or walker), or injuries;

2) have a history of neuromuscular conditions or muscle wasting diseases;

3) are currently participating in physical activity, ≥150 minutes of moderate-to-vigorous physical activity per week (https://www.csep.ca/CMFiles/Guidelines/CSEP_PAGuidelines_older-adults_en.pdf);

4) are not eligible to participate in regular physical activity according to the Canadian Society Exercise Physiology Get Active Questionnaire (https://store.csep.ca/pages/getactivequestionnaire);

5) do not have access to the internet at home via a personal smartphone, tablet (e.g., iPad), or computer.

3.3 Recruitment

Individuals will be recruited to participate in this study via local partner news sources including the electronic and print versions of the Coffee News (http://yourlocalcoffeenews.com/). Study information will also be distributed through social media accounts and posted on partner websites.

3.4 Sample size

A convenience sample of no more than 32 participants will be recruited; participation is limited due to space and camera viewing range restrictions on the Zoom platform. Currently, physiotherapists and kinesiologists at the Physical Activity Centre of Excellence (PACE) are running live online exercise classes for current PACE members and a maximum of 16 participants per class is an optimal number. This number aligns well with previous online exercise pilot studies where the sample size ranges from 10–44 (19, 20).

3. 5 Protocol

An overview of this two-arm randomized pilot trial is depicted in Figure 1. Participants will receive information about the study, complete a brief eligibility screening, complete a medical screening questionnaire (https://store.csep.ca/pages/getactivequestionnaire) and if willing, complete the consent form. After meeting the inclusion criteria and receiving informed consent, participants will complete the baseline data collection. Participants will be asked to wear an accelerometer for a 7d period and complete various questionnaires regarding nutrition, life-space, anxiety, depression, and loneliness during the baseline data collection. Throughout the entirety of the trial, participants will be asked to complete the questionnaires assessing anxiety, depression, and loneliness.

Following baseline data collection, thirty-two participants, sixteen participants/group (8 women and 8 men), will be randomized in a 1:1 ratio to the immediate intervention group or waitlist control group. Participants randomized to the immediate intervention

group will take part in the next exercise program and provide end of study data immediately following the end of the program, and again 8-weeks after taking part in the exercise sessions. Participants assigned to the waitlist control group will receive usual care and will be asked to complete end of study data 8-weeks later. The waitlist control group will then be enrolled in the next available program.

Sessions will take place online thrice-weekly for a total of 8 weeks (total of 24 exercise classes) with a focus on balance, strength and cardio. Sessions will be designed and led by healthcare professionals, physiotherapists and registered kinesiologists to meet the abilities of the participants, via the online platform Zoom. This platform meets McMaster University's privacy legislation and we can effectively create and control content for the proposed trial. Zoom is an US-based person-to-person video-audio platform that can host up to 300 people per user. McMaster has purchased several exclusive licenses for this program, and we have confirmed that we can use up to 20 of those licenses for this research project. Each week participants will be asked to provide brief feedback on the individual session. Eight weeks after the exercise sessions, the co-investigator will contact participants to collect accelerometry data and administer a follow-up questionnaire. A purposeful subsample of participants (N = 24 total, data saturation generally occurs within this range) will be asked to take part in a thirty-minute narrative interview via Zoom to gain a deeper understanding of participants' motivation, attitudes, and beliefs around our exercise class. Using Zoom, we will record and get a high-quality word-for-word transcript (~95% fidelity) of each interview.

Importantly, all planned study measures (except for the accelerometry data, which will be continuous for one 7d period prior to and after the intervention) can be completed in ~45-55min and through computer connections, with assistance provided over the phone if required, and while maintaining physical distancing. All participants will complete a medical screening questionnaire online and with phone assistance if needed. All necessary materials (e.g., accelerometer) will be personally delivered or shipped to participants, via commercial courier service (e.g., Canada Post). Questionnaires will be administered using LimeSurvey (https://research.mcmaster.ca/research-resources/limesurvey/), which is a secure online portal.

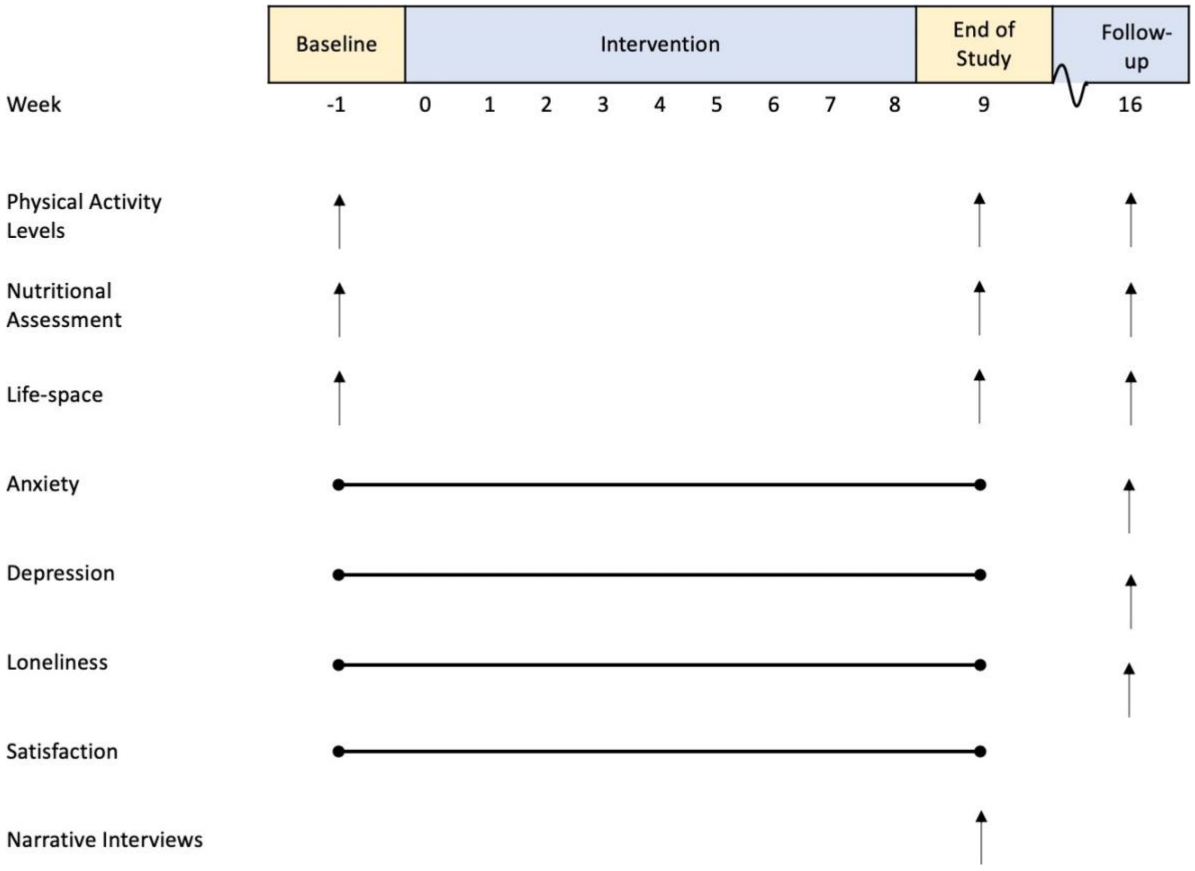

Day	Monday	Wednesday	Friday
**Focus of Exercise**	Strength	Cardio	Balance

Figure 1. A schematic outlining the study design for the present study.

3.6 Outcome measures

Demographic information, including age, sex, education, marital status, and living arrangements will be collected at baseline to describe our study sample. Participants will be asked if they regularly participated in physical activity prior to the Covid-19 pandemic to explore potential differences between those who regularly participated in physical activity and those who do not. However, we will screen participants for those that are, per our exclusion criteria, already performing 150 (or more) minutes per week of moderate to vigorous physical activity.

We will collect and process data to understand program feasibility. Outcomes related to feasibility will be collected throughout the program. These include:

- **Adherence:** Attendance at program sessions will be recorded (people can choose to anonymously participate or screen-share, however, the investigators will know who attends). Satisfaction will be defined as >75% attendance (i.e., 19/24 sessions or greater) at program sessions.
- **Acceptability:** Participant satisfaction and intent to continue participating in physical activity will be collected using questionnaires. Satisfaction will be defined as >60% of participants indicating they were satisfied with the program.

In addition, pertinent measures will be collected at baseline (online), end of program (online), and eight-weeks following the program (online). These include:

- **Accelerometry:** We will use an objective measure using step counts and accelerometry to monitor the impact that our program has on people's activity levels via BodyMedia™ units (21).
- **Life-Space mobility:** We will use the University of Alabama Life Space Assessment, UA-LSA, (22) to assess participants life-space mobility score. It is a global marker of older adults' physical and community mobility and it has been shown to relate to objectively-measured step counts (23).
- **Nutrition assessment:** We will use the Screen II (Seniors in the Community Risk Evaluation for Eating and Nutrition) to determine whether individuals have a potential nutritional problem or are at risk of developing one and identifies those who need further nutrition assessment and treatment (24).
- **Loneliness:** We will use the 11-item Revised University of California Los Angeles loneliness scale (R-UCLA) to assess participants feelings of loneliness (25).
- **Depression:** We will use the Geriatric Depression Scale (GDS-15) (26).
- **Anxiety:** We will use the Geriatric Anxiety Inventory-Short Form (GAI-SF) (27).

3.7 Data analysis

Satisfaction will be determined using *a priori* definitions described above. Failure to achieve satisfaction targets will indicate that modifications are needed to the program design or protocols before moving forward with full implementation.

The analysis will examine the effectiveness of the intervention on individual's step count, nutritional status, life-space mobility score, anxiety, depression, and feeling of loneliness. Descriptive statistics will be calculated for demographic information and expressed as means ± SD for continuous variables and as counts (percent) for categorical variables. Changes in quantitative outcome measures at the end of the program will be analyzed using a two-way repeated measures ANOVA. A p-value <0.05 will be considered statistically significant. Statistical calculations will be performed using IBM SPSS statistical software (version 26).

Qualitative analysis (including data storage, indexing, searching and coding) will be undertaken using NVivo 11 software. Analysis will be based on a mixed (deductive and inductive) approach that draws on a grounded theory approach. We hypothesize that effective uptake of the program amongst older adults cannot be found only in the

literature, but rather in locally grounded experience. This mixed approach will build on existing knowledge while making a place for new emerging ideas.

An initial coding framework will be developed that will constitute the basis for our codebook (deductive approach). We will enrich the coding framework with new themes emerging from the interviews (inductive approach). Once agreement on a coding scheme is reached by the team, one member will code the remaining data. A second member will review the coding for consistency and accuracy. All codes will be mapped and grouped into big themes, based on iterative review by the team, following a content thematic analysis. Data from different categories of participants (identified through quantitative analysis) will be disaggregated during the second step to assess salient differences. Finally, themes will be integrated to generate new interpretations of the diversity of experiences in whether and how the exercise program contributed to physical activity, nutrition, life space, loneliness, anxiety and depression.

4.0 Data monitoring

The main concern for this study data is the presence of anxiety, depression, and feelings of loneliness in the intervention and control groups. Anxiety, depression, and feelings of loneliness may be increased during the Covid-19 pandemic and the GDS-15 questionnaire assesses individuals' symptoms of depression. Therefore, monitoring the score on this questionnaire is important during the study. For this reason, the GDS-15 questionnaire will be checked by the study team while the participants are in the program. If there is a positive score above 5, we will contact the participants' emergency contact and follow-up with both the participant and emergency contact.

This study does not require data and safety monitoring board.

5.0 Potential harms, risks or discomforts

This study poses potential risks and discomforts associated with exercise testing procedures, similar to those associated with any form of physical activity. These include fatigue, fainting, abnormal blood pressure, irregular heart rhythm, and in very rare instances, heart attack or death. Every effort will be made to minimize these potential risks by evaluating preliminary information relating to individual's health and fitness. Participants will be required to complete the Get Active Questionnaire developed by CESP in order to participate in the trial. We will also try to mitigate these potential risks by reiterating the declaration form at the beginning of each exercise class. Additionally, careful observations of the participants during training and testing using video chat (Zoom). To ensure safety of the participants during this study, individuals will be asked to check-in at the end of each class; we will also ensure that the participant has provided us with their home address and an emergency contact. In the event that an abnormal event occurs and/or health is compromised, the moderator on the call will remain on the video call and provide further guidance to the participants, alert the emergency contact and contact emergency services if required.

Additionally, the GDS-15 will be used to manage risk related to symptoms of depression. The study team will check the GDS-15 for a positive score above 5,

indicating the presence of depression and possible suicidal thoughts, weekly to manage risks. There may also be risks involved including anxiety or fatigue when completing the study questionnaires and interviews. In addition, participants allocated to the control group may feel disappointed that they did not receive the intervention. The study investigators interviewing participants and administering intervention/control conditions are healthcare professionals and are qualified to monitor participants in order to assess any risk. All participants, regardless of assigned condition, will continue to have access to usual care. If participants are seen in distress, fatigue or anxious situations, the study investigators will attend to the participant needs and seek out the proper resources and assistance if necessary. The control group will be offered the online exercise class at the end of the wait list.

6.0 Confidentiality

All study personnel will be trained and monitored regularly in the requirement of participants' confidentiality according to research ethics board regulations and following good clinical practice guidelines. All research related procedures including data collection and storage will be carried out on McMaster Universities network, a secure server. No information about any participants will be shared outside of the research team without prior consent unless there are concerns regarding participant health and safety and in this instance these concerns will be communicated to the participant and appropriate healthcare professionals.

7.0 Implications of research

The goal of this pilot study is to determine the satisfaction and potential effects of a live online exercise program for older adults. We hope to expand this pilot to a larger population to explore the adaption of the live online exercise program to a more multicultural population, as well as those living in rural areas. We fully acknowledge that our electronically delivered data collection approach will be a barrier to some older people. Thus, the trial sample will invariably be characterized by participants with higher technological literacy. We plan, however, to develop this concept further by developing, using an end-user co-design approach, an age-friendly usable app that could be made to interface with the preferred platform.

REFERENCES

1. Zhou F, Yu T, Du R, Fan G, Liu Y, Liu Z, et al. Clinical course and risk factors for mortality of adult inpatients with COVID-19 in Wuhan, China: a retrospective cohort study. Lancet. 2020;395(10229):1054-62.
2. Berg-Weger M, Morley JE. Editorial: Loneliness and Social Isolation in Older Adults during the COVID-19 Pandemic: Implications for Gerontological Social Work. J Nutr Health Aging. 2020;24(5):456-8.
3. Patel SS, Clark-Ginsberg A. Incorporating Issues of Elderly Loneliness into the Coronavirus Disease-2019 Public Health Response. Disaster Med Public Health Prep. 2020:1-2.
4. Losada-Baltar A, Jimenez-Gonzalo L, Gallego-Alberto L, Pedroso-Chaparro MDS, Fernandes-Pires J, Marquez-Gonzalez M. "We're staying at home". Association of self-perceptions of aging, personal and family resources and loneliness with psychological distress during the lock-down period of COVID-19. J Gerontol B Psychol Sci Soc Sci. 2020.
5. Goethals L, Barth N, Guyot J, Hupin D, Celarier T, Bongue B. Impact of Home Quarantine on Physical Activity Among Older Adults Living at Home During the COVID-19 Pandemic: Qualitative Interview Study. JMIR Aging. 2020;3(1):e19007.
6. McGlory C, von Allmen MT, Stokes T, Morton RW, Hector AJ, Lago BA, et al. Failed Recovery of Glycemic Control and Myofibrillar Protein Synthesis With 2 wk of Physical Inactivity in Overweight, Prediabetic Older Adults. J Gerontol A Biol Sci Med Sci. 2018;73(8):1070-7.
7. Oikawa SY, Callahan DM, McGlory C, Toth MJ, Phillips SM. Maintenance of skeletal muscle function following reduced daily physical activity in healthy older adults: a pilot trial. Appl Physiol Nutr Metab. 2019;44(10):1052-6.
8. Daskalopoulou C, Stubbs B, Kralj C, Koukounari A, Prince M, Prina AM. Physical activity and healthy ageing: A systematic review and meta-analysis of longitudinal cohort studies. Ageing Res Rev. 2017;38:6-17.
9. Falck RS, Davis JC, Best JR, Crockett RA, Liu-Ambrose T. Impact of exercise training on physical and cognitive function among older adults: a systematic review and meta-analysis. Neurobiol Aging. 2019;79:119-30.
10. Steffl M, Bohannon RW, Sontakova L, Tufano JJ, Shiells K, Holmerova I. Relationship between sarcopenia and physical activity in older people: a systematic review and meta-analysis. Clin Interv Aging. 2017;12:835-45.
11. Stubbs B, Vancampfort D, Rosenbaum S, Firth J, Cosco T, Veronese N, et al. An examination of the anxiolytic effects of exercise for people with anxiety and stress-related disorders: A meta-analysis. Psychiatry Res. 2017;249:102-8.
12. Hill KD, Hunter SW, Batchelor FA, Cavalheri V, Burton E. Individualized home-based exercise programs for older people to reduce falls and improve physical performance: A systematic review and meta-analysis. Maturitas. 2015;82(1):72-84.
13. Gine-Garriga M, Roque-Figuls M, Coll-Planas L, Sitja-Rabert M, Salva A. Physical exercise interventions for improving performance-based measures of physical function in community-dwelling, frail older adults: a systematic review and meta-analysis. Arch Phys Med Rehabil. 2014;95(4):753-69.
14. Miller KJ, Goncalves-Bradley DC, Areerob P, Hennessy D, Mesagno C, Grace F. Comparative effectiveness of three exercise types to treat clinical depression in older adults: A

systematic review and network meta-analysis of randomised controlled trials. Ageing Res Rev. 2020;58:100999.
15. Schrempft S, Jackowska M, Hamer M, Steptoe A. Associations between social isolation, loneliness, and objective physical activity in older men and women. BMC Public Health. 2019;19(1):74.
16. Hwang J, Wang L, Siever J, Medico TD, Jones CA. Loneliness and social isolation among older adults in a community exercise program: a qualitative study. Aging Ment Health. 2019;23(6):736-42.
17. Lindsay-Smith G, O'Sullivan G, Eime R, Harvey J, van Uffelen JGZ. A mixed methods case study exploring the impact of membership of a multi-activity, multicentre community group on social wellbeing of older adults. BMC Geriatr. 2018;18(1):226.
18. Pecanha T, Goessler KF, Roschel H, Gualano B. Social isolation during the COVID-19 pandemic can increase physical inactivity and the global burden of cardiovascular disease. Am J Physiol Heart Circ Physiol. 2020;318(6):H1441-H6.
19. Sinclair Elder JA, Scott SW, Kluge M, Elder LC. CyberEx Internet-Based Group Exercise for Rural Older Adults: A Pilot Study. Activities, Adaptation & Aging. 2016;40(2):107-24.
20. Nikitina S, Didino D, Baez M, Casati F. Feasibility of Virtual Tablet-Based Group Exercise Among Older Adults in Siberia: Findings From Two Pilot Trials. JMIR Mhealth Uhealth. 2018;6(2):e40.
21. Gastin PB, Cayzer C, Dwyer D, Robertson S. Validity of the ActiGraph GT3X+ and BodyMedia SenseWear Armband to estimate energy expenditure during physical activity and sport. J Sci Med Sport. 2018;21(3):291-5.
22. Taylor JK, Buchan IE, van der Veer SN. Assessing life-space mobility for a more holistic view on wellbeing in geriatric research and clinical practice. Aging Clin Exp Res. 2019;31(4):439-45.
23. Tsai LT, Portegijs E, Rantakokko M, Viljanen A, Saajanaho M, Eronen J, et al. The association between objectively measured physical activity and life-space mobility among older people. Scand J Med Sci Sports. 2015;25(4):e368-73.
24. Keller HH, Goy R, Kane SL. Validity and reliability of SCREEN II (Seniors in the community: risk evaluation for eating and nutrition, Version II). Eur J Clin Nutr. 2005;59(10):1149-57.
25. Lee J, Cagle JG. Validating the 11-Item Revised University of California Los Angeles Scale to Assess Loneliness Among Older Adults: An Evaluation of Factor Structure and Other Measurement Properties. Am J Geriatr Psychiatry. 2017;25(11):1173-83.
26. Dennis M, Kadri A, Coffey J. Depression in older people in the general hospital: a systematic review of screening instruments. Age Ageing. 2012;41(2):148-54.
27. Byrne GJ, Pachana NA. Development and validation of a short form of the Geriatric Anxiety Inventory--the GAI-SF. Int Psychogeriatr. 2011;23(1):125-31.
